# Case report: A *de novo* Non-sense *SOX9* mutation (p.Q417*) located in transactivation domain is Responsible for Campomelic Dysplasia

**DOI:** 10.3389/fped.2022.1089194

**Published:** 2023-01-18

**Authors:** Xingxing Qiao, Liping Wu, Jianjun Tang, Rong Xiang, Liangliang Fan, Hao Huang, Yaqin Chen

**Affiliations:** ^1^ Department of Cardiology, Second Xiangya Hospital Central South University, Changsha, China; ^2^ Department of Medical Genetics and Prenatal Diagnosis, Longgang District Maternity and Child Healthcare Hospital, Shenzhen, China; ^3^ Obstetric Inpatient Department, Shenzhen Longgang District Maternity and Child Healthcare Hospital, Shenzhen, China; ^4^ Department of Nephrology, Xiangya Hospital, Central South University, Changsha, China; ^5^ National Clinical Research Center for Geriatric Disorders, Xiangya Hospital, Central South University, Changsha, China; ^6^ Department of Cell Biology, School of Life Sciences, Central South University, Changsha, China

**Keywords:** *SOX9*, transactivation, campomelic dysplasia, terminated mutation, non-sense

## Abstract

**Background:**

Campomelic dysplasia (CD) is an autosomal dominant skeletal dysplasia syndrome characterized by shortness and bowing of lower extremities, and often accompanied by XY sex reversal. Heterozygous pathogenic variants of *SOX9* or rearrangement involving the long arm of chromosome 17 are the causes of disease. However, evidence for pathogenesis of *SOX9* haploinsufficiency is insufficient.

**Methods:**

We enrolled a Chinese family where the fetus was diagnosed with CD. The affected fetus was selected for whole-exome sequencing to identify the pathogenic mutations in this family.

**Results:**

After data filtering, a novel non-sense *SOX9* variant (NM_000346.3; c.1249C > T; p.Q417*) was identified as the pathogenic lesion in the fetus. Further co-segregation analysis using Sanger sequencing confirmed that this novel *SOX9* mutation (c.1249C > T; p.Q417*) was a *de novo* mutation in the affected fetus. This terminated codon mutation identified by bioinformatics was located at an evolutionarily conserved site of *SOX9*. The bioinformatics-based analysis predicted this variant was pathogenic and affected *SOX9* transactivation activity.

**Conclusion:**

CD is a rare condition, which connected with *SOX9* tightly. We identified a novel heterozygous *SOX9* variant (p.Q417*) in a Chinese CD family. Our study supports the putative reduced transactivation of *SOX9* variants in the pathogenicity of CD.

## Introduction

Campomelic dysplasia (CD, OMIM: #114290) is a rare autosomal dominant skeletal dysplasia syndrome caused by rearrangement of 17 chromosome and mutations in *SOX9* gene. The diagnosis is usually made after 20 weeks of pregnancy by ultrasonography characterized by malformation of skeletal including bowing of the legs, especially the tibias, small scapular bones, and cleft palate (1). Otherwise, a small chest, eleven pairs of ribs, micrognathia, flat face, and hypertelorism are also featured. CD is a lethal syndrome due to respiratory distress related to small chest and tracheobronchial hypoplasia (2). In two-thirds of reported 46, XY karyotype affected individuals existed male to female sex reversal or had ambiguous genitalia because of the essential role of *SOX9* in sex determination (3, 4).

*SOX9* (SRY-related HMG-box gene 9, OMIM: 608160), located in chromosome 17q24.3, is an essential transcription factor for both sex and skeletal development. Anomalies of *SOX9* are the main causes of CD, and the rearrangement involving the long arm of chromosome 17 will interrupt the upstream of *SOX9* (5, 6). Most reported cases of CD are caused by intragenic heterozygous mutations in *SOX9* gene, and the deletions of *SOX9* represent strong evidence for the dosage-dependent action of *SOX9* protein in normal chondrogenesis (7, 8).

Heterozygous *de novo* mutations are the main cause of CD and sex reversal. The detected shortness and bowing of tubular lower bones by ultrasonography could lead to the suspect of CD. Here we reported on novel heterozygous terminated mutation in *SXO9* gene (c.1249C > T; p.Q417*) in a CD-affected individual in a Chinese family, and we present new evidence for the pathogenesis of *SOX9* in CD.

## Material and methods

### Ethical compliance

The present study was approved by the Review Board of Shenzhen Longgang District Maternity and Child Healthcare Hospital and was performed in accordance with the principles outlined and enshrined in the Declaration of Helsinki. Written informed consent was obtained from the parents for the publication of any potentially identifiable images or data.

### Participants/patients

The affected fetus and parents were investigated in this study. Amniocentesis was performed to collected the sample of fetus and peripheral blood samples were collected from the parents of fetus. Clinical data are mainly collected from ultrasound measurements and are carefully recorded.

### Whole-exome sequencing

Genomic DNA was extracted using the DNeasy blood and tissue kit (Qiagen, Valencia, CA, United States). The main part of WES was performed at Guangdong Women’s and Children’s Hospital. The filter strategies used are consistent with those outlined in our previous research (9). Briefly, after initial quality control of the data, introns, intergenic and untranslated regions (UTRs), homogeneous single nucleotide variants (SNVs), and variants with a frequency of substitution alleles exceeding 1% in the public databases [1,000 Genomes, dbSNP144, YH database, and Genome Aggregation database (gnomAD)] are first removed and then continue further analyzed. Then, the variants predicted by SIFTm PolyPhen-2, and Mutation Taster as “Disease-causing,” were retained.

## Results

### Clinical description

**Figure 1:**
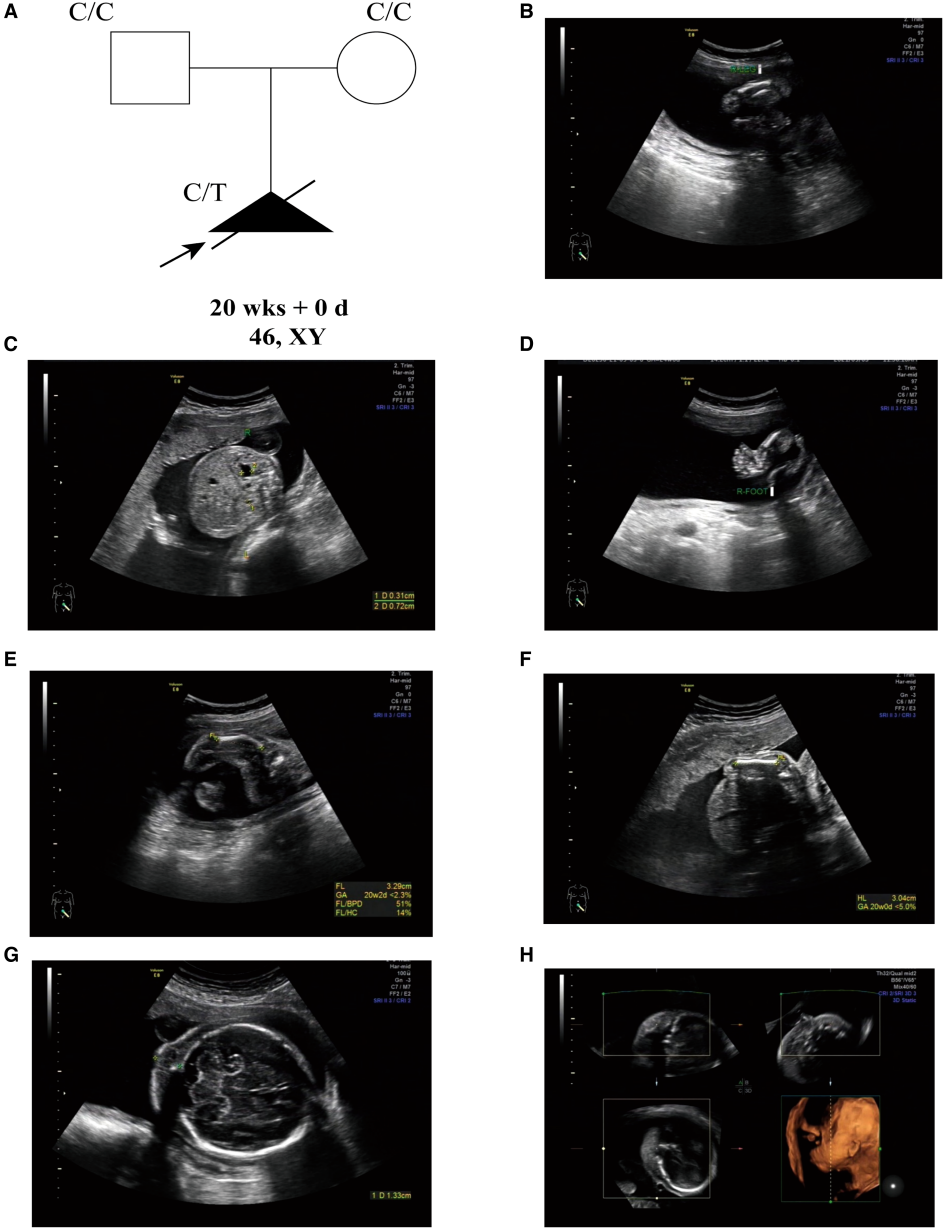
(**A**) the pedigree of this family. Black indicates the affected fetus with CD. White circles are unaffected. Arrow indicates the proband. (**B**) Short femurs. (**C**) Detached right renal pelvis. (**D**) Foot varus. (**E**) Short and curved femurs (<2.5%). (**F**) Shorted humerus (<5%). (**G**) Thickened neck fold (>6 mm). (**H**) Micrognathia.

A 30-year-old, nulliparous woman (G_0_P_0_A_0_) presented in Shenzhen Longgang District Maternity and Child Healthcare Hospital for routine pregnancy prenatal Doppler ultrasound following up and care. The couple has no history of consanguinity, but the gravida has a history of intellectual disability. The initial fetal ultrasonography revealed abnormal long bones in both lower extremities, mild hydronephrosis with detached right renal pelvis, and varus in both feet (Figures 1A–C). Thus, they received further detailed anomaly scan of the fetus. A detailed fetal examination revealed severe malformation of bones, including shortened spine and ribs, leading to a small chest, and the long bones of the fetal limbs are distinctly short and curved, especially the femur. The face of the fetus is seen with micrognathia and significant thickening of the neck fold of 1.3 cm (>6 mm). The amniotic fluid index is 21.7 cm, beyond the normal range of 14.3–15.9 cm. Typical pot-shaped external genitalia was observed in the fetus (Figures 1E–G). After ultrasonography examination, the diagnosis of thanatophoric dysplasia or CD was suspected, and WES was requested for the pathogenesis of the affected fetus.

### Genetic analysis

**Figure 2:**
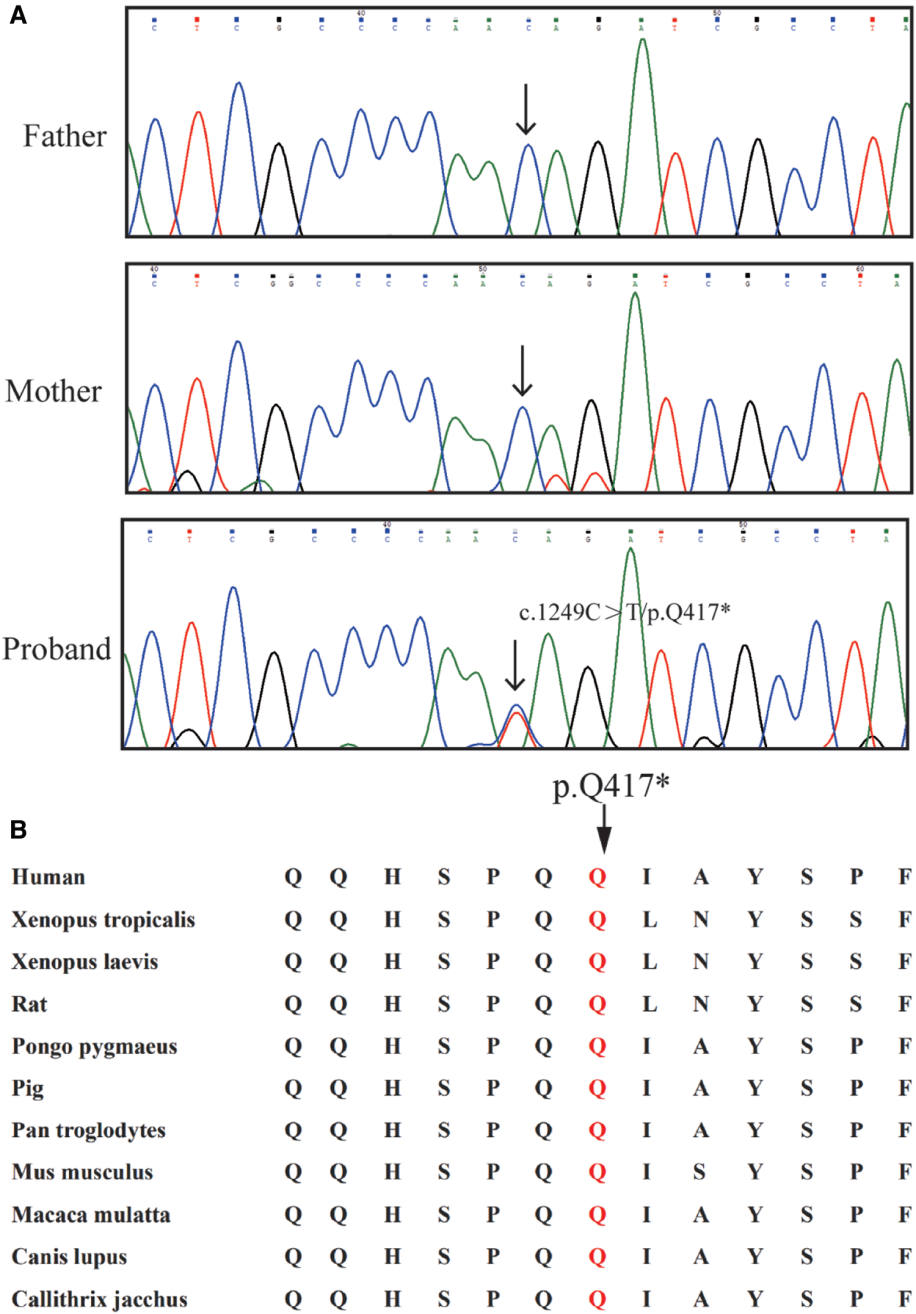
Genetic description of the Chinses family. (**A**) Sequencing results of the *SOX9* mutation. Sequence chromatograms indicate the heterozygosity of the *SOX9* non-sense mutation (NM_000346.3; c.1249C > T; p.Q417*) in the fetus and in the normal parents. Black arrow indicates the mutation site. (**B**) Alignment analysis of this site (p.G285) in COL4A4 amino acid sequences shows that the site (p.417) is highly conserved.

After data filtering, a novel mutation of *SOX9* (NM_000346.3; c.1249C > T; p.Q417*) was highly suspected to be the genetic lesion in the fetus (Figure 2A). No other potential pathogenic mutations were found to cause the malformation of bones. Further co-segregation analysis revealed that the novel *SOX9* mutation did not exist in the parents. This mutation is a *de novo* mutation in this family. Bioinformatics-based prediction revealed that this termination mutation is pathogenic and may affect transactivation of *SOX9*. Otherwise, this novel mutation (c.1249C > T; p.Q417*) was located at a highly evolutionarily conserved site of the *SOX9* protein (Figure 2B).

## Discussion

In this study, a novel mutation (c.1249C > T; p.Q417*) was identified in the affected fetus of a Chinese family by WES and Sanger sequencing. Our results were consistent with previous studies in humans and animals that heterozygous mutations in *SOX9* may lead to CD (10, 11). This mutation occurred within the C-terminus of *SOX9* and was presumably thought to led to a truncated *SOX9* protein and affected the transactivation of *SOX9*. Our study further supports the recommendations released by the International Society for Prenatal Diagnosis, which highlight the application of WES when a fetal anomaly was observed (12).

CD is a lethal, autosomal dominant skeletal dysplasia usually caused by a heterozygous mutation of *SOX9* and often accompanied by sex reversal (4). The *SOX9* protein mainly consists of several domains, including a dimerization domain (DIM), a DNA-binding domain (high-mobility group, HMG), two transactivation domains, and a proline/glutamine/alanine (PQA)-rich domain (13). The missense, nonsense, frameshift, consensus splice site, and loss of function mutations located on one of the three exons of *SOX9* are the main causes of CD (14). Otherwise, the reciprocal translocation involving *SOX9* or its regulatory region also led to CD (15). The reported cases of CD that *SOX9* being completely deletion over the past few decades were thought to represent evidence for the dosage-dependent action of *SOX9* protein in normal chondrogenesis (8, 16). However, Csukasi et al. (14) revealed dominant-negative mutations of *SOX9* in CD-affected individuals, and the reported deletion of *SOX9* also overlapped the upstream enhancer region (7, 17, 18). Thus, it is difficult to confirm the haploinsufficiency of *SOX9* in CD.

The correlation between the type and position of mutations of *SOX9* with resulting phenotype is also lacking. Kwok et al. (19) reported two patients with the same frameshift mutation, but one presented with male phenotype and the other displayed a female phenotype with XY sex reversal. The reported Y440X mutation that presumably resulted in a truncated protein but retains some transactivation function has been described as a less lethal form of CD. The patient carrying the nonsense mutation Q117X has been reported performing well over a 10-year period. However, other reported patients with missense mutations such as Q375X and E400X died in the neonatal period (20).

The CD-causing mutations are usually located in the HGM domain and transactivation domain. *SOX9* gene has two transactivation domains located in the middle (TAM) and the C-terminus (TAC), interacting with transcriptional co-activators or basal transcriptional machinery components. *SOX9* transactivation domains work synergistically or independently of each other to activate chondrocyte-specific genes (13). A recent study revealed an evolutionarily conserved EΦ[D/E]QYΦ motif in TAM, playing a critical role in the transactivation function of *SOX9* protein (21). The missense mutation in EΦ[D/E]QYΦ motif of SOX18 showed impaired transactivation (22). In addition, the interaction of *SOX9* with β-catenin and TRAP230 required TAC, and the reported mutation of TAC (R394G and R437C) which retained normal transactivation of Col2a1 caused testicular dysgenesis without CD and sex reversal, a less lethal result (23, 24). However, the terminated mutation (Q412X) causes a severe phenotype and has a dramatically reduced effect on the *SOX9* activation of Col2a1 (14). In our case, we reported a novel terminated mutation (p.Q417*) of *SOX9*, causing severe and lethal clinical phenotypes, including the leading causes of fetal mortality such as small thoracic, micrognathia, and cleft palate. This mutation is located in the TAC transactivation domain and the terminated mutation p.Q417* presumably resulted in a truncated *SOX9* protein and impaired transactivation. The phenotype caused by mutation p.Q417X located in TAC of *SOX9* was consistent with CD, providing increasing evidence for the correlation between the clinical and radiographic phenotype and the extent to *SOX9* mutations affect transactivation.

In conclusion, there is growing evidence to support the role of *SOX9* in the pathogenesis of CD, but the link between mutations and clinical phenotypes has not been established. Recent studies have shown that the pathogenicity of *SOX9* is related to the activity of transactivation of mutant *SOX9* protein. Severe clinical phenotype is closely related to the reduced activation of Col2a1. Mutations in the two transactivation domains of *SOX9* have received additional attention, and this study provides a truncated mutation located in the TAC domain of *SOX9*, which may affect the transactivation activity of *SOX9*. Our study confirmed the clinical phenotype of this variant. Future research on *SOX9* mutations should pay more attention to the correlation between *SOX9* mutation and transactivation activity.

## Patient perspective

The results of genetic and ultrasound reports supported the diagnosis of CD, and the couple decided to terminate the pregnancy. The parents of the fetus provided written informed consent to participate in this study. Written informed consent was obtained from the couple for the publication of potentially identifiable images or data included in this article.

## Data Availability

'The datasets for this article are not publicly available due to concerns regarding participant/patient anonymity. Requests to access the datasets should be directed to the corresponding author.
